# Idiopathic Urethral Stricture and Nephrogenic Diabetes Insipidus: The Odd Couple

**DOI:** 10.7759/cureus.5076

**Published:** 2019-07-04

**Authors:** Alessandro Maria Berton, Nunzia Prencipe

**Affiliations:** 1 Endocrinology, Diabetology and Metabolism, University of Turin, Turin, ITA

**Keywords:** stenosis, polyuria, polydipsia, deprivation, aquaporin, copeptin

## Abstract

Nephrogenic diabetes insipidus (NDI) is one of the principal defects leading to polyuria-polydipsia syndrome (PPS). In the absence of other evident causes (drug interaction, electrolytic disorders or inherited disease), obstructive uropathy is the most likely aetiology. Direct arginine vasopressin (AVP) assessment during water deprivation test (WDT) remains the gold standard in PPS differential diagnosis despite well characterised limitations in this procedure. A new WDT method using copeptin as reliable surrogate of AVP is proposed. This case represents the first report of an NDI due to idiopathic urethral stricture in an adult and it would like to be explicative of the importance of a correct differential diagnosis of PPS and of the risk related to a prolonged WDT procedure in a frail patient. A 48-year-old male patient presenting with polyuria and polydipsia lasting one month was diagnosed with NDI. Copeptin values were clearly elevated both at baseline and after osmotic stimulus. WDT was complicated by development of acute kidney injury. Abdomen ultrasound demonstrated bilateral hydronephrosis, trabeculated bladder and a residual urine volume of 819 cc, in presence of normal kidney size and prostatic gland. A cysto-urethrography showed a sub-stenosis of 35 mm involving the membranous urethral tract. The patient underwent to balloon dilatation and urethrotomy with complete restitutio ad integrum. In our knowledge, this is the first report of idiopathic urethral stricture complicated by NDI in adult. PPS workup requires a global medical evaluation by an endocrinologist. In the suspicion of NDI, urinary tract obstruction should be considered. WDT remains a cornerstone in the differential diagnosis of PPS and the availability of biomarkers including copeptin may simplify the diagnostic process.

## Introduction

Nephrogenic diabetes insipidus (NDI) is one of the principal defects leading to polyuria-polydipsia syndrome (PPS), together with central diabetes insipidus and primary polydipsia (PP). NDI results from failure of the kidneys to concentrate urine due to a renal insensitivity to the action of the antidiuretic hormone arginine vasopressin (AVP) [1]. Both primary and secondary forms of the disease have been described: primary NDI is principally due to an inherited mutation of vasopressin V2 receptor (AVPR2) or aquaporin-2 (AQP2) genes; whereas secondary NDI is due to lithium treatment, hypercalcemia, hypercalciuria, hypokalemia or obstructive uropathy. In obstructive uropathy, NDI results from suppression of AQP2 expression mediated by increased hydrostatic pressure [2]; missing this diagnosis can lead to end-stage renal disease [3]. Direct AVP assessment during water deprivation test (WDT) is considered the gold standard in PPS differential diagnosis [4]. However, mainly because of the large preanalytical variability of AVP assay, a new WDT method has been proposed using copeptin as reliable surrogate of AVP [5,6]. The following case report represents the first description of an NDI due to idiopathic urethral stricture in an adult. It is also an example of acute kidney injury as a possible WDT side effect.

## Case presentation

A 48-year-old smoker male patient was admitted to our Endocrinology Division for polyuria, mostly nycturia and polydipsia lasting one month. The patient also had urgency urinary incontinence, but not voiding difficulty, temperature or stranguria. Past medical history revealed an untreated atrial flutter (CHADs-VASC 0) and pacemaker implant for sick sinus syndrome; but no history of traumatic brain injury was detected. Family history revealed no significant diseases. Physical examination showed a first-degree obesity (Body Mass Index 32.7 kg/m^2^), mild pretibial bilateral oedema and high heart rate (110 beats per minute). Head, neck, lymph nodes and chest examinations showed no abnormalities. Laboratory data indicated the patient had normal serum electrolyte levels (Na^+^ 141 mmol/L, K^+^ 3.6 mmol/L, Ca^++^ 2.28 mmol/L), normal glycometabolic profile (fasting glucose 79 mg/dl, HbA1c 47 mmol/mol), mild elevation of NT-pro-BNP (1018 pg/ml) of uncertain clinical relevance, normal PSA level (0.38 ng/ml) and progressive increase in serum creatinine (1.45 versus 0.8 mg/dl) in the last year. The hydric balance showed a water intake almost equal to output (6-9 liters per day). Due to suspected diabetes insipidus, plasmatic (p-Osm) and urinary (u-Osm) osmolality evaluations were performed. The analyses showed improperly lower u-Osm levels (121 mOsm/kg), in presence of mild-elevated p-Osm (299 mOsm/kg) with reduced u-Osm/p-Osm ratio (0.4) and urine specific gravity (1.007), in the absence of glycosuria or proteinuria. Thyroid and adrenal deficits were excluded (TSH 2.9 µUI/ml, fT4 11.3 pg/ml, cortisol 148.2 µg/L, ACTH 40 pg/ml). In presence of transient psychiatric agitation symptoms, computed tomography scan was performed to exclude frontal cerebral lesions, and no evidence of encephalic, hypothalamic or pituitary lesions was found.

Following our local clinical standard practice, an indirect WDT followed by a D-amino D-arginine vasopressin (DDAVP) test (4 µg s.c.) was performed (Tables 1, 2).

**Table 1:** Water deprivation test. u-Osm: Urine osmolality; p-Osm: Plasma osmolality; Na+: Serum sodium; NA: Not available; AM: Ante meridiem; PM: Post-meridiem.

Time	0 AM	8 AM	9 AM	10 AM	11 AM	12 AM	1 PM
u-Osm (mOsm/kg)	143	146	147	154	157	164	172
p-Osm (mOsm/kg)	286	297	297	298	297	298	299
Na^+^ (mmol/L)	138	145	144	146	146	145	147
Partial diuresis (cc)	0	+900	+150	+150	+50	+150	+400
Copeptin (pmol/L)	35.4	NA	NA	NA	NA	NA	57.3

**Table 2:** DDAVP test. DDAVP: D-amino D-arginine vasopressin; u-Osm: Urine osmolality.

Time after DDAVP administration	0’	60’	120’
u-Osm (mOsm/kg)	195	196	212
Partial diuresis (cc)	0	+200	+100

According to our local protocol, dehydration test was stopped when serum sodium reached 147 mmol/L and an effective osmotic stimulus was obtained (p-Osm 299 versus 286 mOsm/kg and Na^+^ 147 versus 138 mmol/L; peak versus baseline, respectively) in the absence of significant u-Osm variations (u-Osm 172 versus 143 mOsm/kg; peak versus baseline); thus confirming diabetes insipidus diagnosis. In addition, a lack of response to DDAVP test was observed (u-Osm 195 versus 212 mOsm/kg; basal and post-DDAVP stimulus), suggesting complete NDI. Copeptin values were clearly elevated both at baseline and after osmotic stimulus (35.4 and 57.3 pmol/L, respectively). According to nephrological indication, infiltrating disorders (sarcoidosis, amyloidosis, multiple myeloma, Sjogren’s disease) were excluded.

**Figure 1:**
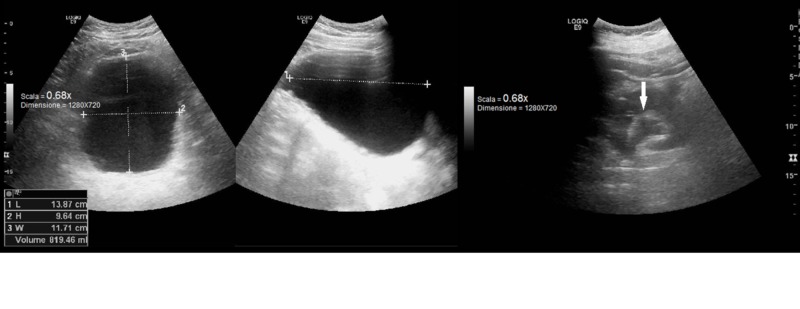
Abdomen ultrasound showing residual urine volume and first degree hydronephrosis (white arrow). L: Length; H: Height; W: Width.

In the following days, serum creatinine levels abruptly increased (3.2 mg/dl; estimated Glomerular Filtration Rate sec. CKD-EPI 22 ml/min/1.73 m^2^) suggesting acute kidney injury (AKI), although appropriate hydration per os was guaranteed. The abdomen ultrasound demonstrated bilateral first degree hydronephrosis, trabeculated bladder and a residual urine volume of 819 cc, in presence of normal kidney size with preserved cortical thickness and normal prostatic gland with central hyperechogenicity. No radiological signs of urinary stones were detected (Figure 1).

**Figure 2:**
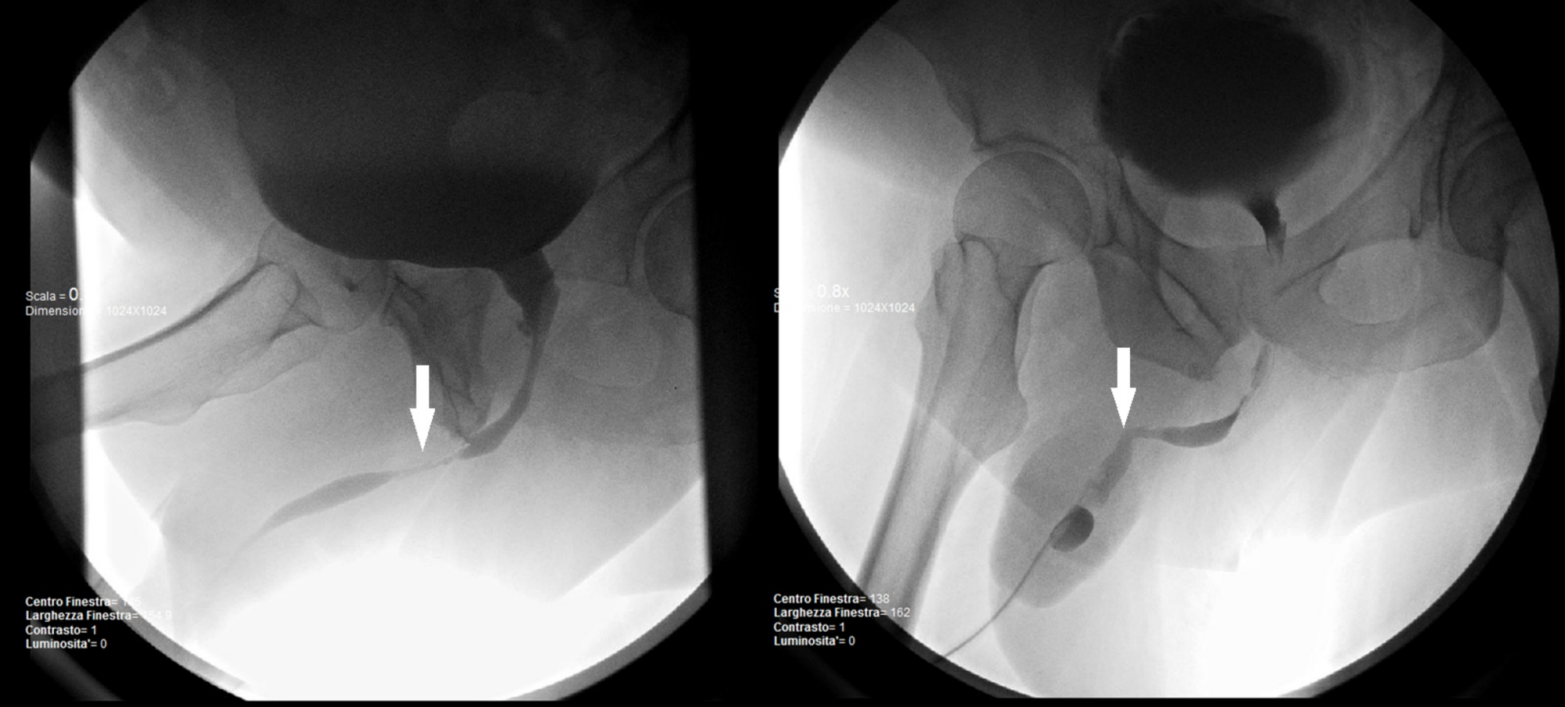
Retrograde cysto-urethrography showing the location, length, and severity of the stricture pre- and post-dilatation procedure (white arrow).

These radiological findings allowed a better definition of the PPS, in which an overflow incontinence was likely hidden by the polyuric state. Therefore, a retrograde cysto-urethrography was performed, highlighting a sub-stenosis of 35 mm involving the membranous urethra tract, with urologic indication for urethrocystoscopy and balloon dilatation. Eventually stricture was resolved with urethrotomy and subsequent long-term urethral catheterization (Figure 2).

One month after catheter removal, a urodynamic study confirmed the resolution of the urinary obstruction and an abdominal ultrasound showed normal residual urine volume in the absence of hydronephrosis. Laboratory analyses demonstrated a restored hydro-electrolytic balance (Na^+^ 140 mmol/L, p-Osm 291 mOsm/kg, u-Osm 377 mOsm/kg, urine specific gravity 1.010, copeptin 15.9 pmol/L) and a normalization of the renal function (creatinine 0.89 mg/dl, estimated Glomerular Filtration Rate sec. CKD-EPI 100.4 ml/min/1.73 m^2^) along with remission of the urinary symptoms and normalization of the water input.

## Discussion

In the absence of other evident causes (drug interaction, electrolytic disorders or inherited disease), obstructive uropathy is the most likely NDI aetiology and a comprehensive urinary tract evaluation is mandatory. Urethral strictures (idiopathic or related to endoscopic procedure, traumatism or inflammatory disease) are not an infrequent cause of obstructive disease (incidence 200-1,200 cases per 100,000 individuals, increasing in people aged ≥55 years) and majority of them are anterior (92.2%) involving the bulbar urethra (46.9%) [7]. In our case, secondary forms have been excluded which to our knowledge represents the first report of idiopathic urethral stricture complicated by NDI in adult.

WDT is the essential tool in differential diagnosis flowchart of PPS and is used to identify the aetiology of the syndrome (primary polydipsia, central or nephrogenic diabetes insipidus), but as known, it requires hospitalization, it is time-consuming (more than 20 hours may be needed), often hard for the patient to tolerate and can lead to severe clinical consequences. Indirect WDT, based on u-Osm variation induced by dehydration first and then by DDAVP administration, is characterized by poor diagnostic accuracy [5]. Direct WDT with AVP measurement is not routinely used in endocrinological practice because of its large preanalytical variability [8]. Copeptin, the C-terminal portion of the AVP precursor, is a stable, surrogate marker that can replace AVP testing. Copeptin is secreted in equimolar levels to AVP by neurosecretory granules of neurohypophysis [9]. Two recent clinical trials have proposed a new WDT method using copeptin as reliable surrogate of AVP [5,6]. This test is demonstrated to be more accurate and, crucially, dehydration would no longer be necessary in NDI identification because basal copeptin value > 21.4 pmol/L detects 100% of polyuric patients affected by AVP insensitivity [6].

In our patient, AKI developed immediately after WDT, suggesting the convenience of a shorter dehydration period or a different diagnostic test. In fact, AKI is a rare but severe complication, which may even require to dialysis in some cases [3]. In this context for example, an initial correct definition of urinary symptoms (overflow incontinence vs urinary urgency), together with basal copeptin determination could already be suggestive for NDI.

Our experience is finally consistent with the recent evidence that copeptin is a valid alternative to WDT and may be especially useful in managing frail patients (e.g., cardiac disease, chronic renal failure, incontinence, psychiatric disorders) and in the elderly. Frequently in these patients prolonged dehydration is not only difficult to be performed, but also unsafe.

## Conclusions

All polyuric-polydipsic patients require a global medical evaluation and the endocrinologist plays the main role in their management. In the suspicion of NDI, all possible obstructive urological causes should be considered in order to be surgical treated preventing further kidney damage. Although direct or indirect WDT remains the cornerstone of the differential diagnosis of the syndrome, a prolonged dehydration could be dangerous in severe polyuric patients. The availability of copeptin may improve and simplify the diagnostic process in these patients, avoiding the WDT practice in NDI.
